# Syndrome of Inappropriate Antidiuretic Hormone Secretion as a Presentation of Untreated Parkinson’s Disease

**DOI:** 10.7759/cureus.37286

**Published:** 2023-04-08

**Authors:** Karim Ali, Yaseen Najjar, Swati Mehta, Geovani Faddoul

**Affiliations:** 1 Internal Medicine, Albany Medical Center, Albany, USA; 2 Internal Medicine/Nephrology, Albany Medical Center, Albany, USA

**Keywords:** parkinson's disease, extrapyramidal symptom, levodopa-carbidopa, syndrome of inappropriate antidiuretic hormone secretion, hyponatremia

## Abstract

Syndrome of inappropriate antidiuretic hormone secretion (SIADH) is the most common electrolyte disorder associated with neurological conditions. Parkinson’s disease (PD) has not been known to be causative of SIADH. We present the case of a 71-year-old male patient with diabetes type II (T2DM) and hypothyroidism admitted with progressive confusion, slow speech, and severe fatigue for one week, accompanied by sluggish body movements for a few months. A neurological exam revealed mild arm rigidity, bradykinesia, resting tremors, and stiff gait. The exam was otherwise normal. Initial blood work showed hypo-osmolar hyponatremia (Na 122 mEq/L, serum osmolarity (Osm) 275 mOsm/kg, and urine Osm 672 mOsm/Kg). CT chest showed localized infiltrate. The initial diagnosis was SIADH secondary to pulmonary process, most probably pneumonia. After starting him on a fluid restriction of 1.5 L/day and urea 15 mg BID, sodium improved gradually to 133 mEq/L on discharge. Urine osmolality continued to be elevated ranging between 700 and 800 mOsm/Kg. An active pulmonary process was ruled out by a pulmonologist. Parkinsonism was diagnosed four weeks after discharge by Neurology who started carbidopa/levodopa. As extrapyramidal symptoms improved, urine osmolality improved as well to 400 mOsm/Kg. Sodium level was maintained between 135 and 137 while urea treatment was stopped and fluid restrictions removed. New-onset SIADH was thought to be secondary to Parkinson’s disease. Parkinson’s disease treatment (carbidopa/levodopa) is known to cause SIADH. In this case, the treatment itself and a dose increase led to improvement in sodium levels and urine osmolality concomitantly with the improvement of the patient’s extrapyramidal symptoms.

## Introduction

Syndrome of inappropriate antidiuretic hormone secretion (SIADH) is a relatively common disorder and one of the most common causes of hyponatremia [1]. It is characterized by euvolemic hyponatremia, low plasma osmolality, normal or elevated natriuresis, and hypouricemia due to the excess release of antidiuretic hormone (ADH) despite decreased or normal osmolarity [2]. SIADH diagnosis and treatment can be challenging due to the multitude of possible etiologies affecting ADH secretion other than serum osmolarity such as pain, medications, lung disease, malignancies, strokes, and cranial surgeries [3]. Parkinson's disease is a progressive neurodegenerative disorder typically presenting in adulthood and due to dopamine depletion from the basal ganglia. It is characterized by motor and nonmotor manifestations such as tremors, bradykinesia and rigidity, depression, sleep disorder, and other cognitive dysfunctions. However, Parkinson's disease (PD) has not been known to be causative of SIADH. We present a case of hyponatremia due to SIADH in the setting of newly diagnosed PD.

## Case presentation

**Figure 1:**
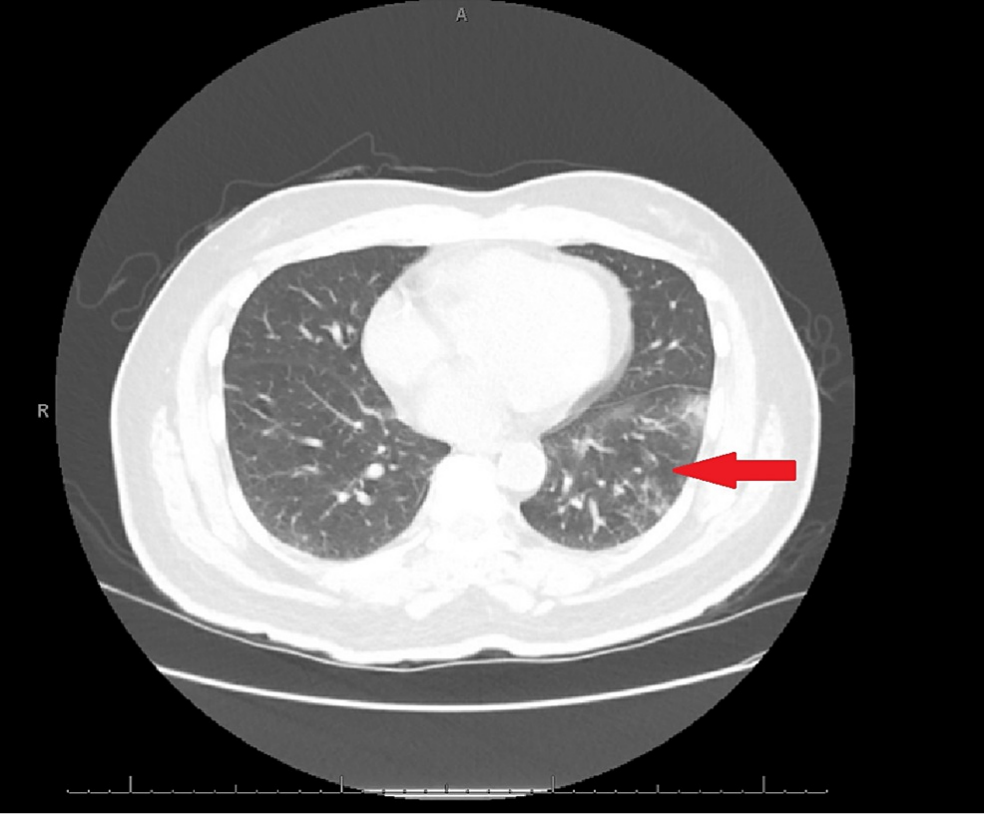
Left lower lobe bronchial inflammatory changes with mild ground-glass opacities

A 71-year-old male patient with T2DM and hypothyroidism, at home on icosapent ethyl, insulin (aspart and glargine), levothyroxine, and rosuvastatin, presented to the hospital with progressive brain “fogginess” (inability to concentrate), slow speech, and severe fatigue over the week before admission. He also reported sluggish body movement and a slow gait for a few months. On admission, he was switched to omega-3 (non-formulary replacement), enoxaparin was added for prophylaxis purposes, and acetaminophen was prescribed PRN for headache/pain. During his exam, the patient had long periods of pausing and a loss of track of conversations. History was provided by the patient’s daughter given his confusion. At baseline, he was independent in his activities of daily living (ADL). The patient denied cough, shortness of breath, abdominal pain, nausea, vomiting or diarrhea, anorexia, polyuria, oliguria, or rashes. The rest of the review of systems was negative. On physical examination, he appeared tired and exhausted but not distressed or in any pain. Auscultation of heart sounds and lungs was normal. The cranial nerve examination was normal. The rest of the neurological exam was limited due to poor interaction. The remaining physical examination was normal. His vitals included a blood pressure of 132/65, heart rate of 65 bpm, temperature of 36.1 Celsius, respirations of 18/min, and a saturation of 99% on room air. On admission, the workup showed hypo-osmolar hyponatremia (sodium 122 meq/l (135 - 145 meq/l), serum osmolarity 275 mOsm, serum uric acid 2.3 mg/dl (3.6 - 8 mg/dl), thyroid-stimulating hormone (TSH) 3.5 mcIU/ml (0.45 - 4.5 mcIU/ml), AM cortisol 24 mcg/dl (4.3 - 22.4 mcg/dl)). To note, we did not have free thyroxine (FT4) measured on admission, but it was measured four months later at 1.3 ng/dl (0.82 - 1.77 ng/dl) with a TSH of 1.02 mcIU/ml while on the same dose of levothyroxine since he was admitted. Urine studies showed (urine sodium: 92 meq/l, osmolarity 672 mOsm). Brain MRI did not show any structural abnormality. CT of the chest (Figure 1) showed mild bronchial wall thickening and peripheral ground-glass density concerning for parenchymal disease in the left lower lobe. Ceftriaxone and azithromycin were prescribed for empirical treatment of possible community-acquired pneumonia. Due to the infiltrate being the only possible cause at the time, a diagnosis of SIADH secondary to pneumonia was made. Empirically, the community-acquired pneumonia antibiotics regimen was given for four days. SIADH was managed by fluid restriction of 1.5 L/day and urea 15 mg BID. Subsequently, sodium improved gradually up to 133 meq/L on discharge.

**Figure 2:**
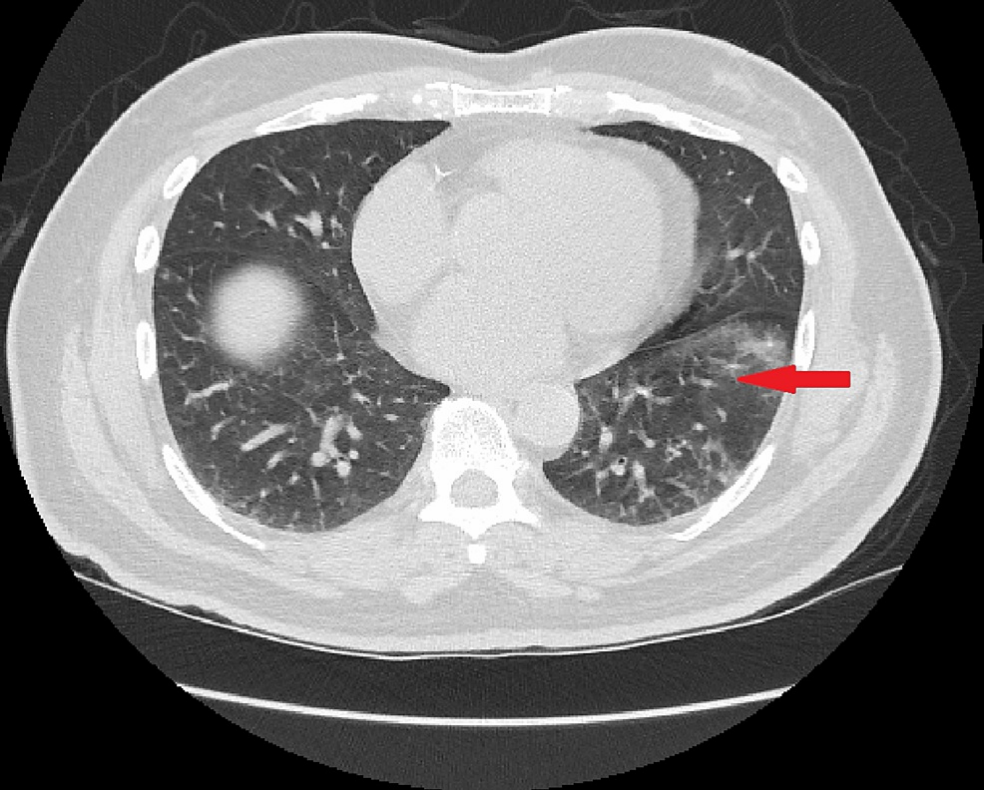
Unchanged appearance of left lower lobe ground-glass opacities and bronchial inflammation. No suspicious malignant lesions

The patient was later seen in the clinic by nephrology, neurology, and pulmonology. Repeated labs showed normal serum Na (133 mEq/L) and elevated urine osmolality (774 mOsm/l). Therefore, the urea solution was continued. During his neurology evaluation, the patient was found to have mild arm rigidity, bradykinesia, resting tremors, and a stiff abnormal gait. He was diagnosed with PD and prescribed levodopa/carbidopa. Thereafter, urine osmolality started to improve from a high of 811 down to 558. Thus, urea was decreased to once a day and then discontinued. Moreover, a repeat CT chest a month after discharge showed a persistent ground-glass appearance of the left lower lung (Figure 2). His pulmonology assessment suggested that the ground glass of the airway was likely atelectasis/fibrosis rather than bacterial or viral pneumonia, especially in the absence of any manifestation such as cough, dyspnea, fever, or leukocytosis. Pulmonary function tests (PFTs) showed mild restrictive pattern forced expiratory volume in one second (FEV1) 64, forced vital capacity (FVC) 69, and FEV1/FVC 0.79. The prodromal Parkinson's disease (PPD) test was negative. An acute pulmonary process was unlikely at this point. Therefore, SIADH was unlikely to be related to the pulmonary process at this point and his hyponatremia was more likely due to untreated PD. After initiating anti-Parkinson’s treatment, the sodium level was successfully maintained between 135-137 while being off urea and fluid restriction. His most recent sodium after one and a half years from the treatment of his PD was 135 meq/l.

## Discussion

**Figure 3:**
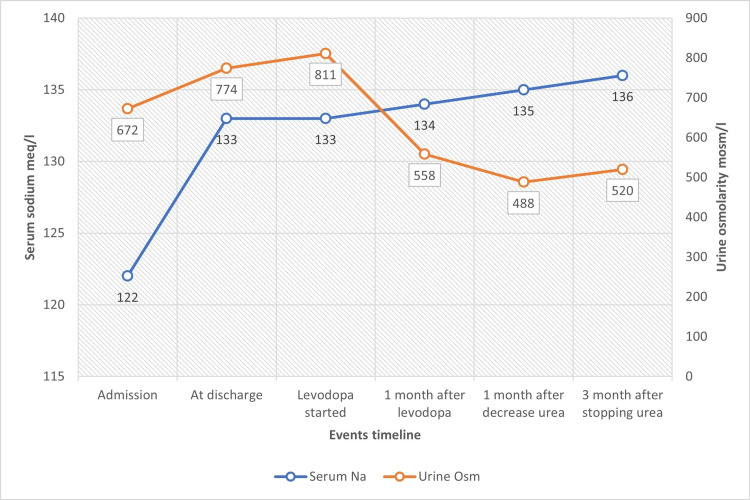
Urine osmolarity and serum sodium trends between admission and post-treatment of Parkinson’s disease

Pulmonary conditions, such as pneumonia, tuberculosis, or obstructive lung disease, for instance, can cause SIADH among other lung-related diseases [4]. In our patient, we could not correlate his lung findings on the CT scan to an active pathology. Brain MRI ruled out structural abnormalities or strokes. A review of his medication list from home did not reveal a common etiology of SIADH. Adrenal insufficiency and hypothyroidism were unlikely given his AM cortisol and TSH levels (Table 1). A plot of his serum sodium and urine osmolarity is presented in Figure 3 to visualize his overall changes from admission to improvement after treatment with levodopa.

**Table 1:** Laboratory results from admission until resolution of the patient’s SIADH S Na: Serum Sodium; S Osm: Serum Osmolarity; U Na: Urine Sodium; Uosm: Urine Osmolarity; TSH: Thyroid-Stimulating Hormone

Date	S Na	S Osm	U Na	U Osm	Uric acid	TSH	Cortisol	Changes
7/23/2021	126	275	92	672	2.6	3.48		Fluid restriction (1.5L/24h)
7/25/2021	130			263			23	Urea 15 mg bid
7/30/2021	133	290	34	774	3.3			None
9/27/2021	135	488						Urea 15 mg down to daily
10/29/2021	137			488				Urea/fluid restriction stopped
3/11/2022	136			520		2.6		No therapy needed

Neurological diseases like cerebrovascular accidents, seizures, traumatic brain injuries, subarachnoid hemorrhage, and other mechanisms affecting the brain can induce SIADH. It is believed that these CNS disturbances can trigger unregulated ADH secretion from stimulation to the hypothalamus regardless of tonicity or volume status. In relation to neurological degenerative diseases, Lewy body dementia has been described in association with hyponatremia through a reset osmostat mechanism [5]. In Parkinson’s disease, dopamine seems to be the link connecting the pathology to SIADH. The direction in which it affects the release of ADH is not clear, however. Dopaminergic agents, such as carbidopa/levodopa and pramipexole, were reported to be associated with the induction of SIADH [6-8]. Tomita et al. reported a case of an 85-year-old woman with Parkinson’s disease who developed a deterioration of consciousness a week after starting pramipexole with laboratory tests confirming the diagnosis of SIADH that rapidly improved after stopping the medication [9].

However, there were no published reports that associate Parkinson’s disease as a direct cause of SIADH such as in our case. After eliminating possible causes of hyponatremia, we could not initially correlate PD to SIADH, as it was not described. In a retrospective view of this case, we know that his initial neurological symptoms were not related to his hyponatremia. The initial correction of his hyponatremia with urea did not improve his symptoms. It was not until later, when treatment for PD was started, that we were able to speculate the correlation with PD. Weaning urea and decrease of fluid restriction was done slowly with a close repeat of his laboratory exams to avoid precipitating severe hyponatremia. As his gait, affect, and energy improved with levodopa, so did his urine osmolarity and his serum sodium. This direct correlation gave us the certainty that his PD was the etiology behind his SIADH and hyponatremia.

## Conclusions

Diagnosing a primary cause of SIADH that becomes chronic can be a challenge. The multitude of etiologies and lack of description of a clear mechanism requires using a broad net of laboratory and imaging tests in the absence of clues from the previously described causative mechanism. SIADH secondary to PD should be recognized as an entity in the differential diagnosis to further facilitate treating patients.
